# MiR-142-3p functions as an oncogene in prostate cancer by targeting FOXO1

**DOI:** 10.7150/jca.41888

**Published:** 2020-01-14

**Authors:** Yi-Fan Tan, Zhi-Yuan Chen, Lei Wang, Min Wang, Xiu-Heng Liu

**Affiliations:** Department of Urology, Renmin Hospital of Wuhan University, Wuhan, Hubei 430060, China.

**Keywords:** Prostate cancer, MicroRNA, MiR-142-3p, FOXO1

## Abstract

Prostate cancer (PCa) is a heterogeneous malignancy, and is a primary cause of cancer-related death in males. Forkhead box transcription factor O1 (FOXO1) exerts antitumor effects in various cancers, including PCa. However, the regulatory mechanism of miR-142-3p on FOXO1 expression in human PCa has not been characterized. In this study, we showed that FOXO1 protein levels were downregulated in PCa tissues and cells. Moreover, FOXO1 expression was a predictor of disease-free survival in patients with PCa and was a predictor of prognosis. Increased expression of FOXO1 suppressed cellular proliferation and induced cell cycle arrest at G0/G1 *in vitro*. However, FOXO1 mRNA and protein levels were inconsistent in human PCa tissues and cell lines. We showed that miR-142-3p levels were negatively correlated with FOXO1 protein levels in PCa. We also showed that miR-142-3p suppressed FOXO1 expression by directly targeting its 3′-untranslated region. Furthermore, suppression of miR-142-3p inhibited cell proliferation and induced cell cycle arrest, and these effects were blocked by FOXO1 knockdown. *In vivo* experiments showed that miR-142-3p knockout impaired tumor growth. Our results validate that FOXO1 acted as a tumor suppressor in PCa and demonstrated that FOXO1 was regulated by miR-142-3p, and miR-142-3p may be a potential target for treatment of PCa.

## Introduction

Prostate cancer (PCa) has become the most common malignancy among men in developed countries, and is the second-leading cause of cancer-related deaths, with an estimated 174,650 newly diagnosed cases and 31,620 deaths in the United States in 2019 1. Over the past decades, although the widespread application of the PSA (prostate specific antigen) test in clinical practice has led to marked reductions in mortality, PSA is an inadequate biomarker of PCa and there is a lack of more specific biomarkers in PCa patients 2, 3. Advances in treatment strategies have led to relatively good outcomes in patients with early stage PCa. However, the prognoses of patients with advanced PCa are poor 4, 5. Therefore, development of novel therapeutic strategies and identification of specific molecular biomarkers for early diagnosis is critical.

Forkhead box transcription factor O1 (FOXO1), encoded by the FOXO1 gene in humans, is a member of the Forkhead transcription factor family, and is widely expressed in living organisms, particularly in the liver, spleen, and lung 6, 7. Forkhead box transcription factor O1 has been identified as an important transcriptional effector in the insulin and insulin-like growth factor 1 (IGF-1) signaling pathways 8, 9. In addition, FOXO1 is involved in numerous cellular functions, such as the cell cycle, apoptosis, and metabolism 10. Recent studies have shown that decreased FOXO1 protein was closely linked to onset and progression of many types of human cancer, which indicated that FOXO1 may exert anti-tumor activity and may be a promising therapeutic target 11-14. However, the regulatory mechanism of FOXO1 expression in human PCa remains to be fully explored.

MicroRNAs (miRNAs) are endogenous, short, non-coding, single-stranded RNA molecules comprised of 19 to 25 nucleotides 15. These molecules negatively regulate target gene expression through post-transcriptional regulation, typically by binding to the 3′-untranslated region (3′UTR) of mRNAs 16. MicroRNAs have been reported as biomarkers for detection and diagnosis of tumors, including PCa 17, 18. In addition, studies have shown that miRNAs may be closely associated with disease onset and progression 19. For example, miR-217 acted as an oncogene by promoting proliferation and migration of bladder cancer through KMT2D 20. Studies of gastric cancer showed that miR-338-3p promoted cell apoptosis and attenuated migration via inhibition pf PTP1B 21. Moreover, miR-142-3p has been shown to promote cell proliferation in human nasopharyngeal carcinoma by repressing SOCS6 22. However, the potential function of miR-142-3p in human PCa, and the association between FOXO1 and miR-142-3p, requires further investigation. In the present study, we showed that FOXO1 exerted anticancer effects through inhibition of cell proliferation and induction of cell cycle arrest in PCa cells. Moreover, we showed that miR-142-3p was a direct upstream regulator of FOXO1 in PCa. Our results suggested that miR-142-3p may function as an oncogene and may be a potential target for treatment of PCa.

## Material and Methods

### Patient samples

A total of 20 PCa specimens with paired normal tissues were collected from patients who had undergone prostatectomy at Urologic department, Renmin Hospital of Wuhan University between 2016 and 2018. None of the patients had undergone chemotherapy or radiotherapy prior to the operation. Informed consent was gathered from the patients prior to the operation. This study was approved by the Institutional Ethics Committee of Renmin Hospital of Wuhan University and strictly followed the guidelines of the Institutional Ethics Committee.

### Cell lines and transfection

Three PCa cell lines (PC3, DU145, and LNCAP) and a human prostate epithelial cell line (RWPE1) were purchased from the American Type Culture Collection (Manassas, USA). Prostate cancer cell lines were maintained in RPMI-1640 culture media (Gibco) supplemented with 10% heat-inactivated fetal bovine serum (Gibco) and 1% streptomycin-penicillin. Human RWPE1 cells were cultured in complete medium containing K-SFM (keratinocyte serum-free medium, Gibco). All cells were maintained in an incubator with 5% CO_2_ at 37 ˚C. MicroRNA 142-3p inhibitors and mimics, and control RNA, were designed and synthesized by Ribobio Company (Guangzhou, China). DU145 cells were transfected with 50 nM of miR142-3p mimics, and LNCAP cells were transfected with 50 nM of miR142-3p inhibitors. Transfection was performed using Lipofectamine 2000 according to the manufacturer's instructions (Invitrogen, USA). After 48 h, cells were harvested for further analyses.

### Data mining

The GEPIA 2 database was used to investigate the expression of FOXO1 mRNA in various types of cancer (http://gepia2.cancer-pku.cn). StarBase version 3.0 was used to review miR-142-3p expression in prostate cancer (http://starbase.sysu.edu.cn/panCancer.php) 23, 24.

### Western blotting analysis

Cells and tissues were collected and lysed with RIPA buffer containing phenylmethanesulfonylfluoride (Beyotime, China). Equivalent amounts of total protein were separated by 10% SDS-PAGE (Servicebio, China), then transferred to PVDF membranes (Servicebio, China). The membranes were then blocked in TBST containing 5% nonfat milk, and the membranes were incubated with primary antibodies overnight at 4 °C. The membranes were then incubated with secondary antibodies. An ECL kit (Multisciences, China) was used to detect proteins. Primary antibodies against FOXO1 (2880) and cyclin D1 (2978S) were purchased from Cell Signaling Technology. Antibodies against p21 (ab109520) and GAPDH (ab9485) were purchased from Abcam.

### Immunohistochemistry

Tissues were fixed in 4% paraformaldehyde, embedded in paraffin, then sliced into 4.0-μm sections. Subsequently, sections were deparaffinized, rehydrated, and incubated with primary antibodies over night at 4°C. Following incubation, the sections were washed three times with PBS and incubated with secondary antibodies. Finally, all sections were stained with 3,3-diaminobenzidine (Servicebio, China) and visualized using a light microscope (Olympus, Japan). The primary antibodies used for immunohistochemistry were anti-FOXO1 (2880, CST, USA) and anti-KI67 (ab15580, Abcam). The average integrated optical density (IOD) was determined for quantitation using Image-Pro Plus software (Version 6.0). The relative mean IOD of each group was divided by that of the control group.

### Cell proliferation assay

Prostate cancer cells were seeded in a 96-well plate and incubated overnight. The cells were then transfected with a FOXO1-overexpressing plasmid or FOXO1 siRNA, and a miR-142-3p mimic or a miR-142-3p inhibitor. Ten microliters of CCK8 reagent (Dojindo, Japan) was added to each well, and the cells were incubated at room temperature in the dark for 2 h. The absorbance was measured at 450 nm using a spectrophotometer. GraphPad Prism 7 was used to determine the cell proliferation (GraphPad Software, Inc., San Diego, USA).

### Flow cytometry analysis

Cell suspensions were harvested using 0.25% trypsin and transferred to precooled 70% ethanol and incubated at -20 °C overnight to fix the cells. The cells were treated with RNA enzyme and propidium iodide, then incubated in the dark for 30 min. A flow cytometer (BD Biosciences, USA) was used to analyze the samples, and the data were processed using ModFit LT 2.0 software.

### Plasmids and RNA interference

The FOXO1 overexpression plasmids or control plasmids were designed and constructed by GenePharma Company (Shanghai, China). For overexpression analysis, DU145 cells were infected with overexpression plasmids using Lipofectamine 2000 according to the manufacturer's instructions (Invitrogen, USA). Small interfering RNAs that targeted sequences of FOXO1, and negative controls, were purchase from GenePharma Company (Shanghai, China). The targeted sequences were as follows: FOXO1 sense, 5'-CCAUGGACAACAACAGUAATT-3'; and antisense, 5'-UUACUGUUGUUGUCCAUGGTT-3'. LNCAP cells were cultured in 6-well plates and transfected with siRNA targeted to FOXO1 using Lipofectamine 2000 according to the manufacturer's instructions (Invitrogen, USA). Cells were harvested and RNA and protein were extracted at 48 h post-transfection.

### Quantitative real-time PCR

Total RNA from tissues and cell lines was extracted using TRIzol regent (Invitrogen, USA). Complementary DNA was synthesized using the TaqManH MicroRNA reverse transcription kit (ABI, CA) or PrimeScript RT Reverse transcriptase reagent kit according to the manufacturer's instructions (Takara). Quantitative real-time PCR (qRT-PCR) analysis was performed using the SYBR Premix Ex Taq kit (Takara) and a StepOnePlus Real-Time PCR System (Applied Biosysterms, USA). Levels of miR-143-3p and FOXO1 were normalized to U6 and GAPDH, respectively. The 2-ΔΔCt method was used to determine the relative expression of genes. The target gene primers were designed by Shanghai Sangon Biotech Co., Ltd (Shanghai, China) and as follows: miR-142-3p (forward): 5′-GTCGTATCCAGTGCAGGG-3′; miR-142-3p (reverse): 5′-CGACGTGTAGTGTTTCCTA-3′; FOXO1 (forward): 5′-TGTCCTACGCCGACCTCATCAC-3′; FOXO1 (reverse): 5′-GCACGCTCTTGACCATCCACTC-3′; U6 (forward): 5′-CTCGCTTCGGCAGCACA-3′; U6 (reverse): 5′-AACGCTTCACGAATTTGCGT-3′; GAPDH (forward): 5′-CAACGTGTCAGTGGTGGACCTG-3′; GAPDH (reverse): 5′- GTGTCGCTGTTGAAGTCAGAGGAG-3′.

### Luciferase reporter assay

The FOXO1 3′UTR luciferase reporter vector was designed and constructed by Shanghai Sangon Biotech Co., Ltd (Shanghai, China). DU145 and LNCAP cells were transiently transfected with miR-142-3p mimic or miR-142-3p inhibitor, respectively, and with FOXO1 3′UTR luciferase reporter and Renila control luciferase vector using Lipofectamine 2000 transfection reagent (Invitrogen, USA). After 48 hours, the cells were collected and lysed. Dual Luciferase Reporter Assay System (Promega, USA) was used to measure luciferase activity.

### Animal experiments

All animal experiments were approved by the Animal Care and Use Committee of Wuhan University. Male BALB/C nude mice (4-5 weeks old, Beijing Vital River Laboratory Animal, China) were housed under specific pathogen-free conditions. LNCAP cells transfected with miR-142-3p inhibitor or NC were prepared by suspending 5×10^6^ cells in 100 µL of PBS. The cell suspensions were subcutaneously injected into the left flanks of the nude mice (n=5 per group). Tumor volume was evaluated every 5 days using Vernier calipers and calculated using the following equation: 0.5 × length × width^2^ (mm3). After 30 days the mice were sacrificed and the tumors were excised.

### Statistical analysis

Statistical analysis was conducted using SPSS 20.0 software (SPSS Inc, USA). Experiments were performed in triplicate. The data are presented as means ± SD. Student's t-test and analysis of variance were used to determine statistically significant differences. Differences were considered statistically significant when P < 0.05.

## Results

### Downregulation of FOXO1 in PCa tissue specimens and cell lines

**Figure 1:**
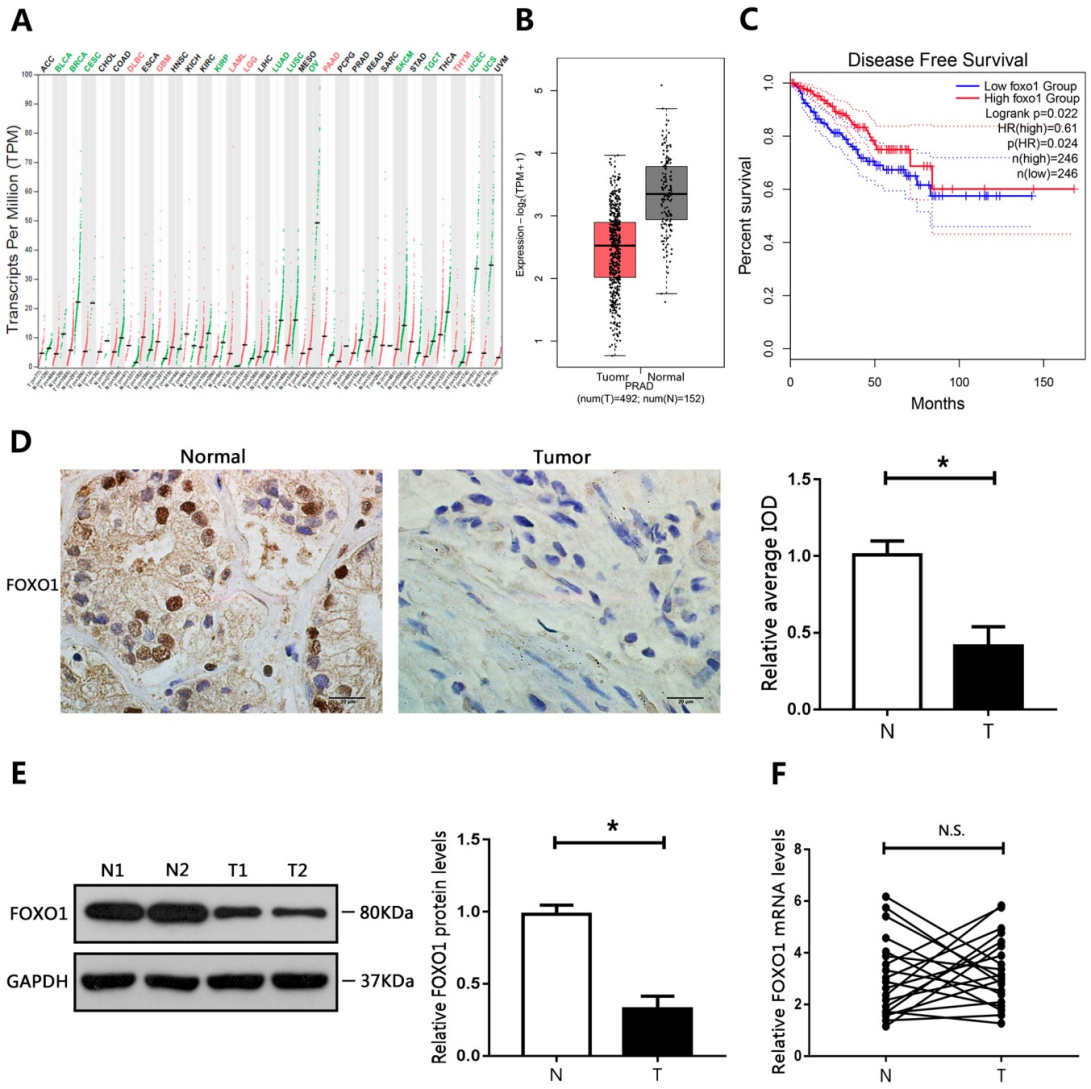
Downregulation of FOXO1 in PCa tissue specimens. **(A)** Expression of FOXO1 in various human tumor tissues and normal tissues. **(B)** Expression of FOXO1 in human cancerous and paired normal prostate tissues. **(C)** Kaplan-Meier curve of disease-free survival.** (D)** Immunohistochemical staining of FOXO1 in PCa specimens and paired normal tissues. Image-Pro Plus software was used to determine the average IOD values. **(E, F)** Messenger RNA and protein levels of FOXO1 were determined in PCa tissues and paired normal tissues. The bar graph shows relative levels of FOXO1. *P < 0.05 vs. normal tissues.

**Figure 2:**
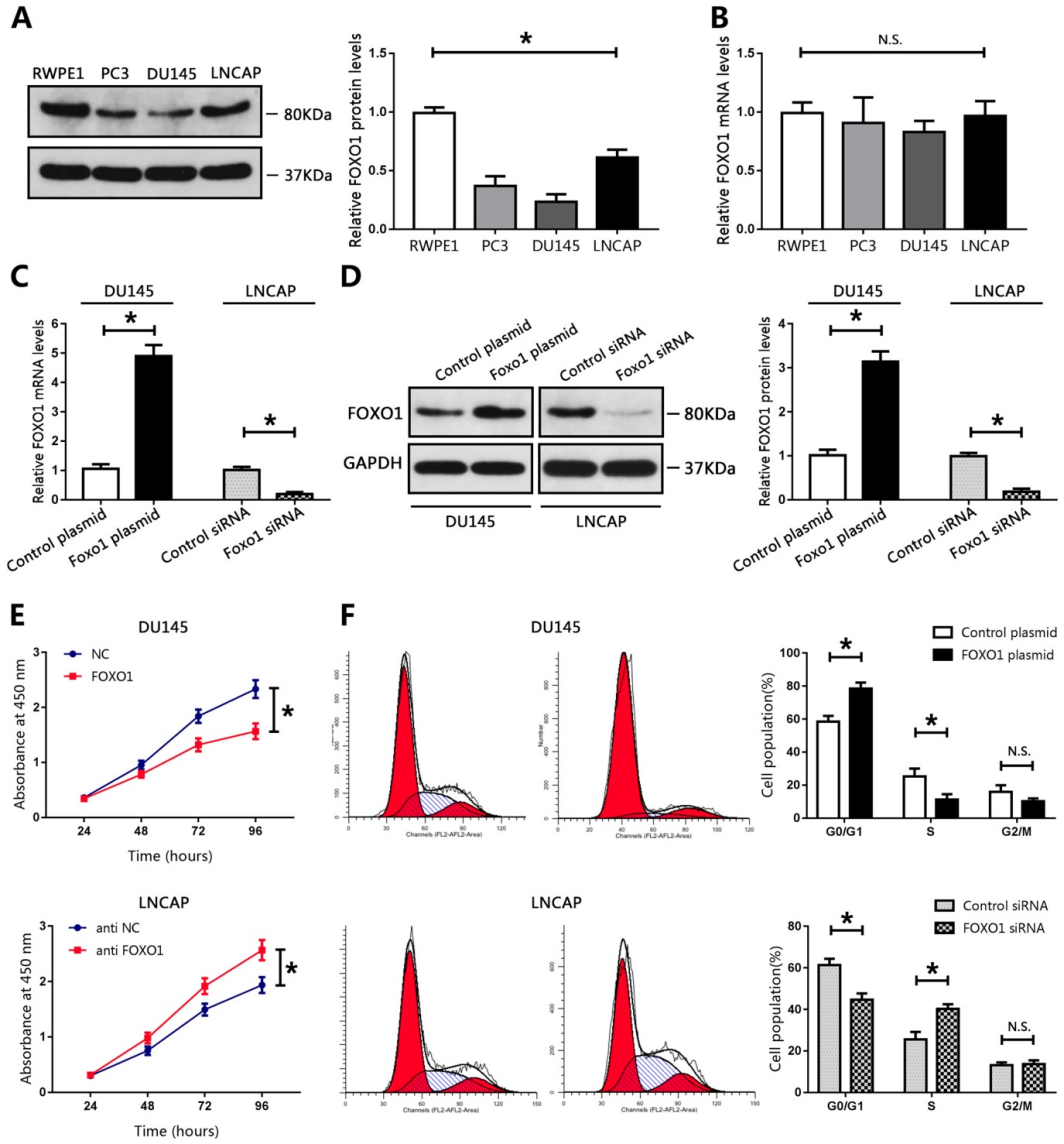
FOXO1 functioned as a tumor suppressor in PCa cell lines. **(A, B)** Levels of FOXO1 protein and mRNA in PCa cells and RWPE1 cells, and bar graphs from three independent experiments. *P < 0.05 vs. RWPE1 cells. **(C-F)** DU145 cells were transfected with FOXO1 plasmid or control plasmid, and LNCAP cells were transfected with FOXO1 siRNA or control siRNA. **(C,D)** Transfection efficacy was evaluated using RT-qPCR or western blotting. The bar graph represents three independent experiments. **(E)** Cell proliferation was measured using CCK8 assay. **(F)** Cell cycle was evaluated using flow cytometry. *P < 0.05 vs. control group.

The GEPIA database was used to evaluate the expression of FOXO1 in different cancers. The results showed that FOXO1 levels were decreased in many cancer types (Figure 1A). In addition, FOXO1 gene expression in PCa was mined in TCGA (The Cancer Genome Atlas) using the GEPIA database. The results showed that FOXO1 was downregulated in PCa tumor tissue compared to corresponding surrounding normal tissues (Figure 1B). To further determine whether FOXO1 could be a prognostic marker for PCa, Kaplan-Meier survival analysis and the log-rank test were performed. The results from the GEPIA database showed that low levels of FOXO1 predicted reduced disease-free survival and were associated with poor prognosis (Figure 1C). We then evaluated the levels of FOXO1 in PCa specimens. Immunohistochemical staining and western blotting showed that FOXO1 protein levels were decreased in PCa tissues relative to those in adjacent normal tissues (Figure 1D, E). However, RT-qPCR results showed no significant differences in FOXO1 mRNA levels between PCa and adjacent normal tissues (Figure 1F). We then evaluated FOXO1 levels in PCa cell lines. Consistent with the results in human PCa tissues, FOXO1 protein levels were downregulated in PCa cell lines compared with those in RWPE1 cells, but FOXO1 mRNA levels were not significantly different between PCa and RWPE1 cells (Figure 2A, B).

### FOXO1 functioned as a tumor suppressor in PCa cell lines

We evaluated the biological function of FOXO1 in PCa cell lines. Compared with those in RWPE1 cells, the levels of FOXO1 protein in PCa cell lines, particularly in DU145 cells, were markedly decreased (Figure 2A). The levels of FOXO1 were only moderately decreased in LNCAP cells compared to those in RWPE1 cells (Figure 2A). Therefore, DU145 and LNCAP cells were used for subsequent analyses. A FOXO1-overexpressing plasmid vector and control vector were established and transduced into DU145 cells. Real-time PCR and western blot data showed that FOXO1 levels were increased in DU145 cells (Figure 2C, D). Furthermore, siRNA targeted to FOXO1 and control siRNA were constructed and transfected into LNCAP cells, and RT-PCR and western blot were used to evaluate transfection efficiency. The results indicated that FOXO1 levels were markedly decreased in LNCAP treated with siRNA targeted to FOXO1 compared to those treated with control siRNA (Figure 2C, D). We then used the CCK-8 assay to evaluate cell proliferation in PCa cells. Overexpression of FOXO1 in DU145 cells significantly inhibited cell proliferation compared with DU145 treated with the control vector, and si-FOXO1 promoted cell proliferation in LNCAP cells (Figure 2E). These results showed that FOXO1 suppressed PCa cell proliferation. We also examined the effects of FOXO1 expression on cell cycle progression. The results showed that FOXO1 overexpression strongly induced cell cycle arrest at G0/G1 in DU145 cells, and knockdown of FOXO1 in LNCAP cells promoted cell cycle progression at S phase (Figure 2F). These results indicated that FOXO1 exerted anti-cancer effects in PCa cells.

### MiR-142-3p was a direct upstream regulator of FOXO1

**Figure 3:**
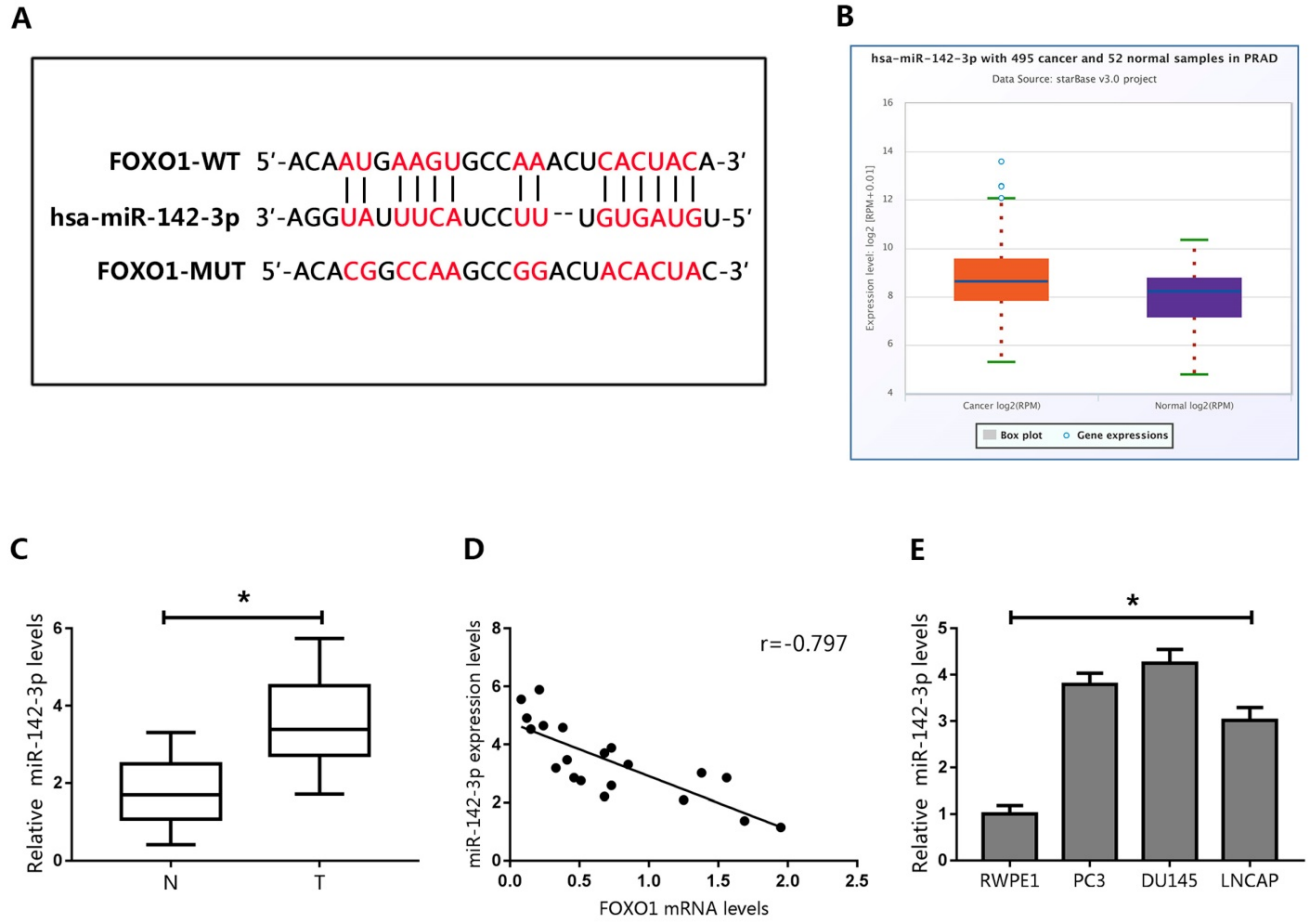
Prediction of FOXO1 as a target of miR-142-3p. **(A)** Potential miRNA-142-3p binding sites on the FOXO1 3′-UTR.** (B)** Expression of miR-142-3p in human PCa tumor tissues and normal tissues. **(C)** Expression of miR-142-3p in human cancerous and paired normal prostate tissues. *P < 0.05 vs. normal tissues. **(D)** Negative correlation between the expression of FOXO1 and miR-142-3p in human PCa specimens. **(E)** MicroRNA 142-3p levels in PCa cells and RWPE1 cells. *P < 0.05 vs. RWPE1 cells.

The expression levels of FOXO1 mRNA and protein were inconsistent in human PCa tissues and cell lines. Therefore, we hypothesized that FOXO1 protein levels in PCa could be regulated by post-transcriptional modifications. The role of miRNAs in post-transcriptional regulation has been extensively studied. Therefore, we predicted that the stability of FOXO1 protein might be regulated by miRNAs. Three databases (TargetScan, PicTar, and StarBase) were searched to identify miRNAs that could target FOXO1. Each database predicted that miR-142-3p could directly bind to FOXO1 at its 3′UTR (Figure 3A).

To evaluate the potential association between FOXO1 and miR-142-3p, miR-142-3p levels were examined in the StarBase database. The data showed that miR-142-3p levels were elevated in PCa tissues (Figure 3B). We then evaluated miR-142-3p expression in human PCa specimens, and showed that miR-142-3p expression was upregulated in PCa tissues (Figure 3C). Pearson's correlation analysis showed that levels of miR-142-3p negatively correlated with FOXO1 expression (Figure 3D).

**Figure 4:**
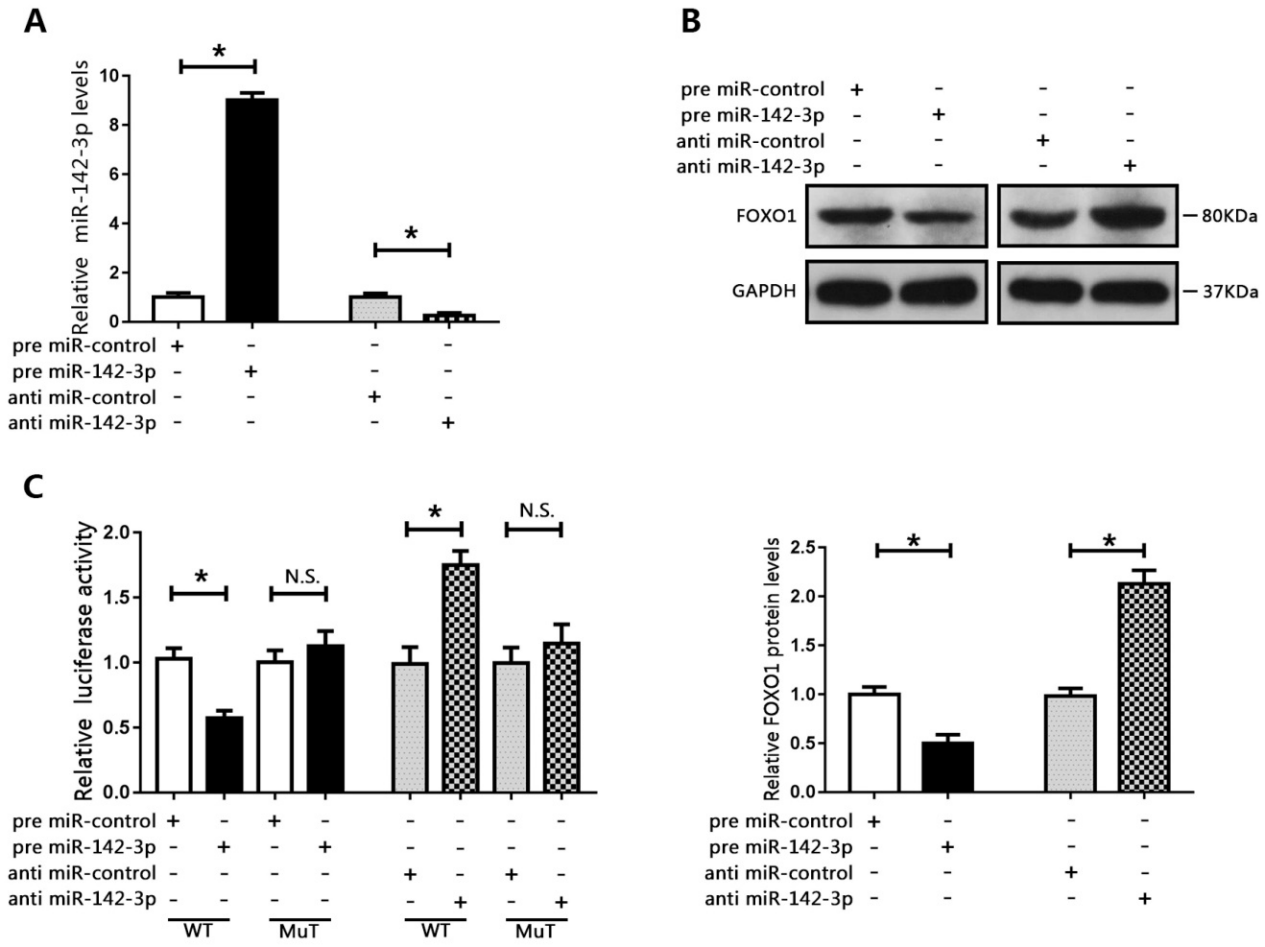
FOXO1 was a target of miR-142-3p.** (A)** Transfection efficacy was evaluated using RT-qPCR following transfection of DU145 cells with miR-142-3p mimics and transfection of LNCAP cells with miR-142-3p inhibitors. *P < 0.05 vs. control.** (B)** The expression of FOXO1 was analyzed by western blotting and RT-qPCR. *P < 0.05 vs. control.** (C)** Luciferase activity in DU145 cells co-transfected with FOXO1-WT or FOXO1-MuT and miR-142-3p mimics, and in LNCAP cells co-transfected with FOXO1-WT or FOXO1-MuT and miR-142-3p inhibitors. *P < 0.05 vs. control.

We then examined the expression of miR-142-3p in PCa cells. The results showed that miR-142-3p was upregulated in PCa cells compared to those in RWPE1 cells (Figure 3E). Interestingly, miR-142-3p levels were moderately increased in LNCAP cells, but were substantially increased in DU145 cells. Therefore, we transfected DU145 cells with miR-142-3p mimics and LNCAP cells with miR-142-3p inhibitors. Real time qPCR analysis showed that the expression of miR-142-3p was substantially enhanced in DU145 cells and repressed in LNCAP cells (Figure 4A). We then evaluated the expression of FOXO1 following transfection with miR-142-3p mimics or inhibitors. The data showed that increased miR-142-3p expression resulted in decreased FOXO1 protein levels in DU145 cells, and inhibition of miR-142-3p expression in LNCAP cells resulted in increased FOXO1 protein levels (Figure 4B). To further characterize the interaction between miR-142-3p and FOXO1, luciferase reporter vectors containing putative binding sites in the 3′UTR of FOXO1 were constructed. The results of luciferase reporter assay showed that increased expression of miR-142-3p inhibited luciferase activity of FOXO1 with the wild-type, but not the mutated vector. In contrast, suppression of miR-142-3p markedly enhanced FOXO1 luciferase activity with the wild-type reporter vector, but luciferase activity was not altered for the FOXO1-Mut vector (Figure 4C). These results indicated that FOXO1 was a direct target of miR-142-3p.

### MiR-142-3p promoted PCa cell proliferation and cell cycle progression by targeting FOXO1

**Figure 5:**
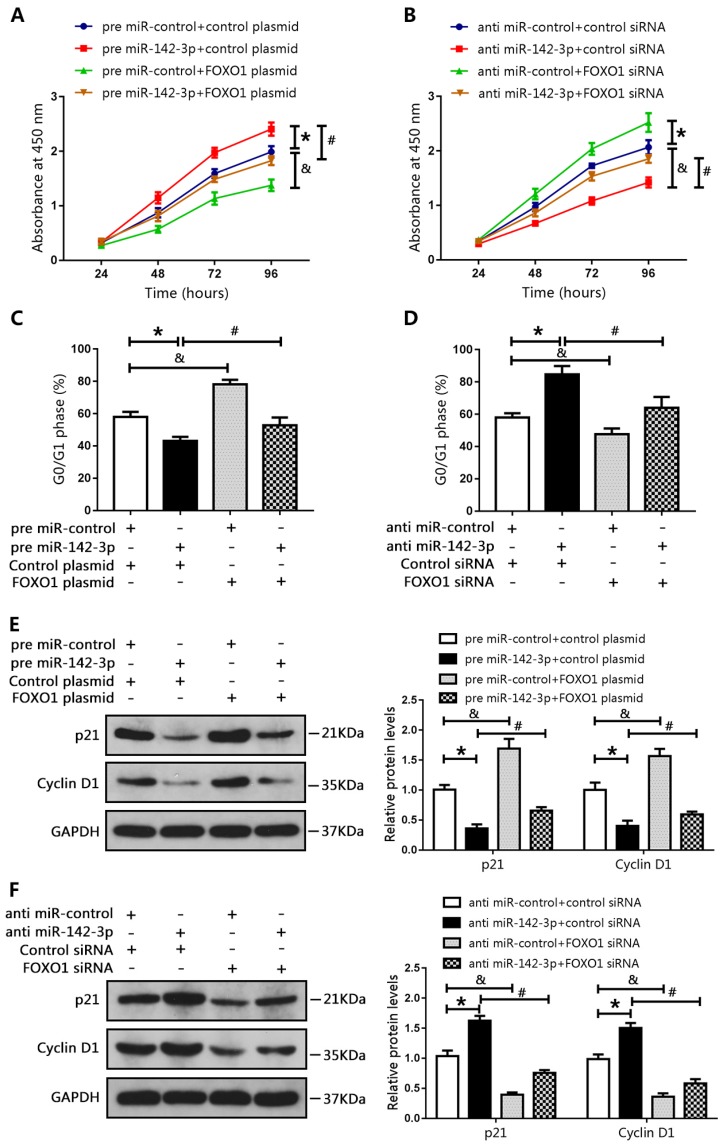
MicroRNA 142-3p facilitated PCa cell proliferation and cell cycle progression by repressing FOXO1. DU145 cells were co-transfected with miR-142-3p mimics and FOXO1 plasmid, and LNCAP cells were co-transfected with miR-142-3p inhibitors and FOXO1 siRNA. **(A, B)** Cell proliferation was analyzed using CCK8 assay. **(C, D)** Cell cycle arrest at the G0/G1 phase was detected using flow cytometry.** (E, F)** Protein levels of P21 and Cyclin D1 were evaluated by western blotting, and bar graphs from three independent experiments are presented. *P < 0.05 vs. NC; #P < 0.05 vs. pre miR-142-3p+ control plasmid or anti-miR-142-3p+ control siRNA. &P < 0.05 vs. NC.

We co-transfected DU145 cells with a miR-142-3p mimic and a FOXO1-overexpression plasmid, and co-transfected LNCAP cells with a miR-142-3p inhibitor and FOXO1 siRNA. Results of CCK8 assay showed that miR-142-3p overexpression promoted cell proliferation. However, this effect was blocked by increased FOXO1 expression (Figure 5A). In addition, FOXO1 siRNA reduced the inhibitory effect of anti-miR-142-3p on LNCAP cell proliferation (Figure 5B). Cell cycle analysis showed that the number of DU145 cells in the G0/G1 phase was decreased following transfection with the miR-142-3p mimic, and this effect was blocked by increased FOXO1 expression (Figure 5C). Furthermore, inhibition of FOXO1 expression using siRNA notably blunted miR-142-3p inhibition-induced cell cycle arrest at G0/G1 (Figure 5D). Western blot analysis showed that the protein levels of p21 and cyclin D1 were decreased following transfection with the miR-142-3p mimic, and this effect was blocked by increased expression of FOXO1 (Figure 5E). Transfection with FOXO1 siRNA blocked miR-142-3p inhibitor-mediated upregulation of p21 and cyclin D1 (Figure 5F). These results showed that miR-142-3p promoted cell proliferation and cell cycle progression by targeting FOXO1.

### MiR-142-3p regulated tumor growth *in vivo*

**Figure 6:**
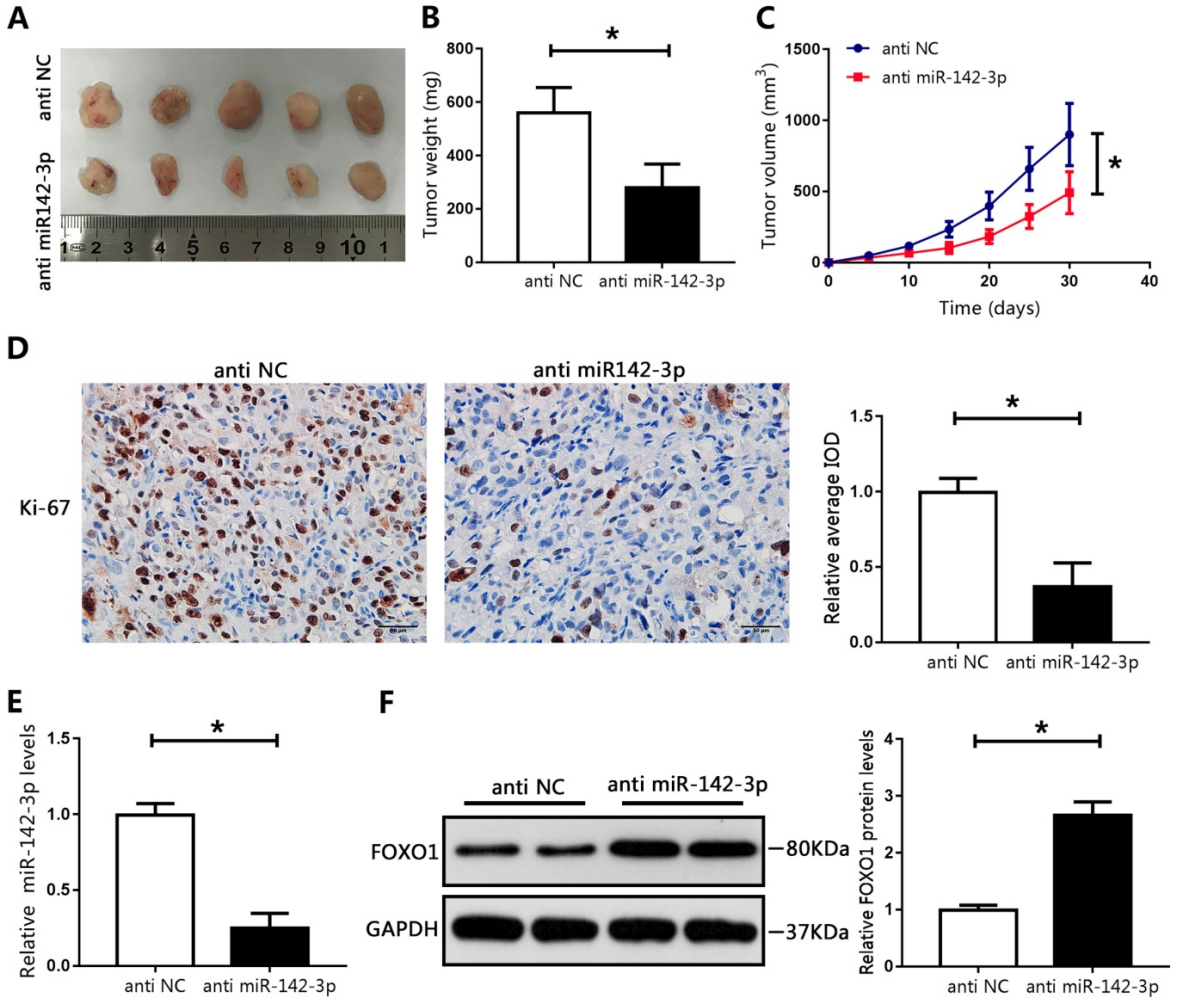
MicroRNA 142-3p regulated tumor growth *in vivo.* **(A)** Image of tumors collected from mice. **(B)** Weights of tumors were analyzed. **(C)** Tumor volume curve. **(D)** Immunohistochemical analysis of Ki-67 in xenografted tissues. Image-Pro Plus software was used to determine the average IOD values. (E) The expression of miR-142-3p was measured using RT-qPCR. **(F)** The levels of FOXO1 were determined by western blotting. *P < 0.05 vs. anti-NC.

To recapitulate the findings of *in vitro* experiments, the effects of miR-142-3p were evaluated *in vivo*. LNCAP cells transfected with miR-142-3p inhibitor were subcutaneously injected into male nude mice. Compared with those in the NC group, the tumor weights and volumes in the miR-142-3p knockdown group were significantly reduced (Figure 6A-C). Immunohistochemical staining showed a notable decrease in levels of Ki-67 in the miR-142-3p knockdown group compared with those in the NC group, as indicated by IOD values (Figure 6D). Furthermore, RT-qPCR analysis showed that miR-142-3p mRNA levels were reduced in the miR-142-3p knockdown group (Figure 6E). Western blotting showed that FOXO1 expression was significantly enhanced in the miR-142-3p knockdown group relative to that in the NC group (Figure 6F). These results showed that suppression of miR-142-3p impaired PCa tumor growth *in vivo*.

## Discussion

The process of progression of PCa to advanced stages is unclear, and the underlying molecular mechanisms of this progression require further investigation. Many studies have shown that dysregulation of FOXO1 was associated with onset and progression of various cancer types 13, 25. For example, FOXO1 was reported to inhibit tumor sphere formation capacity of gastric cancer cells through regulation of LGR signaling 13. The expression of FOXO1 was downregulated in hepatocellular carcinoma cell lines, and restoration of FOXO1 expression suppressed cell metastasis through inhibition of the promoter activity of ZEB2 25. Our previous study showed that upregulation of FOXO1 through BRD4 inhibition suppressed tumor cell proliferation, promoted apoptosis, and induced cell cycle arrest in PCa 26. Moreover, FOXO1 was downregulated in bladder cancer, which was associated with poor outcomes 27. Consistent with these findings, the present study showed that FOXO1 levels were markedly decreased in PCa tissue samples, and positively correlated with disease-free survival in patients with PCa. These findings indicated that low levels of FOXO1 may have contributed to onset of PCa, and that FOXO1 might function as a tumor suppressor in PCa. In addition, increased expression of FOXO1 *in vitro* significantly inhibited cell proliferation and induced cell cycle arrest. Furthermore, decreased FOXO1 expression facilitated cell proliferation *in vitro*. Interestingly, the expression levels of FOXO1 mRNA and protein were inconsistent in some human cases of PCa, which suggested that regulation of FOXO1 protein expression in PCa may involve post-transcriptional modification.

MicroRNAs, which are important post-transcriptional regulators, have been shown to be involved in tumorigenesis 28. In this study, bioinformatics analysis indicated that miR-142-3p might be an upstream regulator of FOXO1. MicroRNA 142-3p, generated from the miR-142 hairpin and located at chromosome 17q22, is involved in various pathological and physiological processes, such as various human cancers, and formation and differentiation of hematopoietic stem cells 29-31. MicroRNA 142-3p plays multiple roles in human cancers. Previous studies showed that miR-142-3p functioned as a tumor suppressor and was downregulated in breast cancer tumors, and increased expression of miR-142-3p suppressed cell viability and metastasis through repression of Bach-1 29. In contrast, miR-142-3p was upregulated in non-small-cell lung cancer and functioned as an oncogene, and overexpression of miR-142-3p promoted cell proliferation through inhibition of TGFβ1 expression 30. Our study showed that miR-142-3p was upregulated in PCa tissues and cell lines relative to non-tumor samples and normal prostate cells. Moreover, we showed that miR-142-3p levels were negatively correlated with FOXO1 in PCa, and confirmed that miR-142-3p repressed FOXO1 expression through binding to the 3′UTR of FOXO1 mRNA.

Our study showed that miR-142-3p overexpression facilitated cell proliferation and inhibited FOXO1 protein expression, and knockdown of miR-142-3p inhibited cell proliferation and tumor growth in xenograft mouse models and increased FOXO1 protein expression. Furthermore, we showed that increased expression of FOXO1 abrogated miR-142-3p-induced cell proliferation, which suggested that miR-142-3p promoted cell proliferation by targeting FOXO1. Other studies have shown that p21 directly interacted with cyclin D1 and induced CDK4 and CDK6 expression, which resulted in inhibition of G1/S progression 32. In this study, we found that p21 and cyclin D1 levels were decreased following administration of miR-142-3p mimics, and this effect was blocked by upregulation of FOXO1, which indicated that miR-142-3p facilitated cell cycle progression through FOXO1. These findings showed that miR-142-3p may function as a tumor promotor in PCa through repression of FOXO1. However, the mechanism by which miR-142-3p expression is elevated in human cancer remains has not been characterized.

In conclusion, our study showed that FOXO1 exerted anti-tumor effects in PCa through inhibition of cell proliferation and induction of cell cycle arrest. These effects may have resulted from negative regulation of FOXO1 by miR-142-3p. Therefore, the miR-142-3p-FOXO1 axis might be a potential therapeutic target for treatment of PCa.
